# A new histone deacetylase inhibitor remodels the tumor microenvironment by deletion of polymorphonuclear myeloid-derived suppressor cells and sensitizes prostate cancer to immunotherapy

**DOI:** 10.1186/s12916-023-03094-0

**Published:** 2023-10-25

**Authors:** Zude Chen, Xiaoshuang Yang, Zugen Chen, Minzhao Li, Wei Wang, Riwei Yang, Zuomin Wang, Yuxiang Ma, Yulong Xu, Shan Ao, Leqi Liang, Chao Cai, Changning Wang, Tuo Deng, Di Gu, Hongqing Zhou, Guohua Zeng

**Affiliations:** 1 Department of Urology and Guangdong Key Laboratory of Urology, The First Affiliated Hospital of Guangzhou Medical University, Guangzhou, China; 2 Athinoula A. Martinos Center for Biomedical Imaging, Department of Radiology, Massachusetts General Hospital, Harvard Medical School, Charlestown, MA USA; 3 Department of Plastic Surgery, The Affiliated Hospital of Zunyi Medical University, Zunyi, China; 4 The Second Ward of Urology, Qujing Affiliated Hospital of Kunming Medical University, Qujing, China

**Keywords:** HDAC inhibitor, Epigenetics, RM1 cell line, MyC-CaP cell line, Prostate carcinoma, Anti-PD-1, Bone metastatic PCa

## Abstract

**Background:**

Prostate cancer (PCa) is the most common malignancy diagnosed in men. Immune checkpoint blockade (ICB) alone showed disappointing results in PCa. It is partly due to the formation of immunosuppressive tumor microenvironment (TME) could not be reversed effectively by ICB alone.

**Methods:**

We used PCa cell lines to evaluate the combined effects of CN133 and anti-PD-1 in the subcutaneous and osseous PCa mice models, as well as the underlying mechanisms.

**Results:**

We found that CN133 could reduce the infiltration of polymorphonuclear myeloid-derived suppressor cells (PMN-MDSCs), and CN133 combination with anti-PD-1 could augment antitumor effects in the subcutaneous PCa of allograft models. However, anti-PD-1 combination with CN133 failed to elicit an anti-tumor response to the bone metastatic PCa mice. Mechanistically, CN133 could inhibit the infiltration of PMN-MDSCs in the TME of soft tissues by downregulation gene expression of PMN-MDSC recruitment but not change the gene expression involved in PMN-MDSC activation in the CN133 and anti-PD-1 co-treatment group relative to the anti-PD-1 alone in the bone metastatic mice model.

**Conclusions:**

Taken together, our work firstly demonstrated that combination of CN133 with anti-PD-1 therapy may increase the therapeutic efficacy to PCa by reactivation of the positive immune microenvironment in the TME of soft tissue PCa.

**Supplementary Information:**

The online version contains supplementary material available at 10.1186/s12916-023-03094-0.

## Background

ICB therapy targeting PD-1 or PD-L1 elicits durable antitumoral effects in various cancer types [1, 2]. However, high immune-refractory rates have been observed in the prostate cancer with use of single-agent ICB [3], even when combinational treatment with CTLA-4 [4]. Tumors nonresponsive to single-agent ICB therapy is due to deficient of infiltrating T cells or an increased immunosuppressive TME by myeloid cells such as MDSCs. Specially, PMN-MDSCs can suppress cancer-killing cytotoxic mediator secretion in CD8 + T cells through the arginase-1 (Arg-1) and inducible nitric oxide synthase (iNOS) expression [5]. Indeed, previous studies have provided evidence that treatment with ICB such us ipilimumab can significantly increase tumor-infiltrating T cells in patients with prostate cancer in the TME [6]; therefore, depleting the immunosuppressive cells may be critical to improve the effect of immunotherapies. HDAC inhibitors (HDACIs) which can suppress epigenetic modifiers enzyme had anti-tumor activity. Recently, it has been reported that HDACIs combined with ICBs therapy resulted in positively synergy efficacy by upregulated function of positive immunocytes and ablate negative immunocytes in preclinical or clinical trials [6–9]. However, as far as we known, there was not any data about the anti-tumor efficacy involved the combination ICB with HDACIs in PCa.

In this study, our data explored the impact of our new HDACi, CN133, on the prostate cancer when combined with PD-1 in in vivo models of prostate cancer. CN133 showed a higher permeability across BPB (blood-prostate barrier) when compared to SAHA and entinostat. Our data showed that combination therapy of CN133 with anti–PD-1 significantly reduced tumor mass compared to either agent alone in the subcutaneous murine prostate cancer models. CN133 decreased tumor growth by suppressive PMN-MDSC infiltration in TME and altered the polarity of their immunosuppressive character to a nonfunctional phenotype. Mechanistically, CN133 could reduce the secretion of Pro-MDSCs accumulation cytokines. However, combination treatment did not reduce tumor mass in the osseous PCa compared with single-agent ant-PD-1 treatment. Gene expression of treated tumors in the bone marrow demonstrated expression of functional molecules involved in PMN-MDSC activation did not alter in the CN133 combination with anti-PD-1 group relative to the anti-PD-1 alone.

In summary, our data suggested that CN133 can alter suppressive TME through decreased PMN-MDSC recruitment and its function in prostate cancer, and CN133 combined with ICB therapy can delay tumor growth significantly. However, a limited response of bone metastases prostate cancer to ICB and CN133 combination therapy suggest a synergic epigenetic target with ICB may engender minimal anti-tumor activities in the bone metastases prostate cancer period.

## Methods

### Compound

CN133 were synthesis from Professor Changning Wang, Massachusetts General Hospital, Harvard Medical School, Boston, USA. The equipment and procedures chosen to produce and identify CN133 were consistent with the literature as described previously [10, 11].

### Experimental animals

One hundred twenty 6-week-old male rats were purchased from Guangdong medical laboratory center, and 6-week-old male BALB/c nude mice (*n* = 12), C57BL/J6 mice (*n* = 52), and FVB mice (*n* = 52) were obtained from the Animal Care Unit of Guangdong, China (the experimental sample size is predicted from the estimated effect size and level of variability). All of animal experiments were performed in the laboratory of the First Affiliated Hospital of Guangzhou Medical University. All animals were housed in cages of 485 × 300 × 200 mm (groups of 2 for rat, groups of 5 for mice) and raised in suitable living conditions; all experiments were performed on the basis of the National Institutes of Health Guide for the Care and Use of Laboratory Animals. All animals were equally spaced into different groups according to the random number provided by the supplier; more details are in Additional file 1: Table S1. We set accidental death before the cut-off time as an exclusion criterion and no animals were excluded from this study. All mice were anesthetized with 2% isoflurane inhalation and executed by using the cervical dislocation method. Only the designer was aware of the grouping and treatment of the experiment, and the experimenter took measurements on samples at complete randomness.

### Human prostate cancer specimens

Prostate tumor tissue samples were collected as part of an institutional review board-approved protocol at the First Affiliated Hospital of Guangzhou Medical University. This study included 9 cases of PSA (prostate-specific antigen) unresponsive PCa patients and 7 cases of PSA responsive PCa patients after anti-PD-1 treatment, who having received prior hormone treatment with confirming mCRPC by radiographic evidence of metastases, and biochemical progression. PSA response was defined with complete response (PSA < 0.2 ng/ml) or partial response (PSA decline ≥ 50% from baseline) during 6 months treatment with pembrolizumab 200 mg intravenously every 3 weeks (Additional file 1: Table S2).

### LC/MS/MS

Six weeks rats (*n* = 120) were divided into 3 groups (5 mg/kg CN133, 5 mg/kg entinostat, 5 mg/kg SAHA) at eight time points. The plasm and prostate tissue of rat were collected at eight time points (0.5 h, 1 h, 2 h, 4 h, 6 h, 8 h, 12 h, 24 h).

Prostate tissue preparation: 10 mg of prostate tissue samples, add 800 μL methanol and vortex for 2 min, grind at 60 Hz for 90 s and sonicate at 4 °C for 30 min, stand at − 20 °C for 1 h, centrifuge at 12000 rpm at 4 °C for 15 min, and then take 200 μL for the test.

Plasma sample preparation: take 100 μL of sample into a 2-mL centrifuge tube, add 300 μL of methanol, vortex for 2 min; grind at 60 Hz for 90 s; sonicate at 4 °C for 30 min before standing at − 20 °C for 1 h; and then centrifuge at 12,000 rpm for 15 min at 4 °C; take 200 μL for further calculations finally.

Detection of samples: drugs were diluted with 10% methanol aqueous solution to obtain four standard solution concentrations (50 ng/mL, 100 ng/mL, 500 ng/mL, and 2000 ng/mL) to prepare a standard curve. Using the standard curve, the MRM multi-ion scanning mode in LC–MS/MS mass spectrometry was applied to detect and calculate the drug concentration in each sample.

### Animal models

RM1 prostate cancer cells (1 × 10^6^, obtained from the American Type Culture Collection) were inoculated subcutaneously into the right flank of BALB/c nude mice (*n* = 12), C57BL/J6 mice (*n* = 52), and FVB mice to perform subcutaneous tumor of MyC-CaP cell line (*n* = 40) or injected into the right bone marrow to build PCa models of bone metastases (*n* = 12), respectively. When the tumor volume reached 100 mm^3^ (the tumor volume was determined with the formula: length × width^2^ × 0.52), CN133 was administered intraperitoneally twice a week at 1 mg/kg twice per week. Antibodies were injected intraperitoneally at 10 mg/kg twice a week including anti-mouse PD-1 antibody (clone: RMP1-14-CP162; Bioxcell) and mouse IgG2a (clone: N/A-CP150; Bioxcells).

### Immunofluorescent Staining of patients or mouse tumors

Paraffin-embedded sections were de-paraffinized in xylene and rehydrated using alcohol. Citrate buffer were applied to retrieve antigen by incubating the sections by boiling for 10 min in the microwave. Before incubation with primary antibodies at room temperature for an hour, cells were fixed with 4% paraformaldehyde for 10 min and then rinsed with PBS twice and incubated 20 min with fluorescence-conjugated secondary antibodies. The nuclei staining was located with DAPI (Invitrogen) and coverslipped. Immunofluorescent images were first exported and quality by Akoya system. Tumor cryosections were stained with anti-CD3 (clone: D4V8L; cell signaling technical, Mouse), anti-CD8 (clone: D4W2Z; cell signaling technical, Mouse), anti-CD11b (clone: D6X1N; cell signaling technical, Human, Mouse), anti-Ly-6G (clone: E6Z1T; cell signaling technical, Mouse), anti-CD15 (clone: MC480, Human), and anti-CD33 (clone: EPR23051-101, Abcam, Human). Tissues were quantified with Mantra Quantitative Pathology Workstation.

### Intra-femoral injection to build bone metastatic prostate cancer mouse models

Briefly, the FVB mice were anesthetized with isoflurane. For intra-femoral injection, after depilating and sterilizing, ports through the middle of the patellar ligament were inserted by a 30-G syringe needles into the bone marrow cavity. Then, 2 × 10^5^ RM1 prostate cancer cells expressing the luciferase gene in 20 μL PBS was slowly injected into the bones.

### Bioluminescence imaging

In vivo bioluminescence imaging (BLI) was performed weekly with PerkinElmer IVIS lumina III. Briefly, bone metastatic FVB mice were anesthetized with isoflurane. Mice were immediately placed onto the bed of IVIS and imaged, after administration of 100 μL 30 mg/ml D-luciferin (PerkinElmer, 122799) intraperitoneally. The IVIS data were analyzed by calculation over the bone region using Living Image software (PerkinElmer) and normalized with total count/second to remove the disturbance of exposure duration.

### MRI imaging of bone PCa mouse models

For magnetic resonance imaging (Bruker BioSpin, Billerica, MA), the animals were placed on the animal bed to scan in both coronal and axial planes after anesthetized with isoflurane. The tumor volumes were assessed using ImageJ program.

### Flow cytometry and fluorescence-activated cell sorting

Prepared for single cell suspensions: for the murine samples, subcutaneous tumor mice or bone tumor mice were sacrificed by CO_2_. Blood samples were drawn from tumor mice heart, and tissues of spleens, femurs, and tumors were extracted and minced by scissor. Femurs and tumors need to be digested with collagenase IV (Sigma-Aldrich, 0.2%) for 30 min at 37 °C and were terminated by RMPI 1640 (serum). Suspensions was strained through a 70-μm cell strainer to obtain a single-cell fluid. Red blood cells were lysed by lysis buffer (BD, 555899).

Antibody incubation and examination: 1 × 10 ^6^ cells from single cell suspensions were labeled with fluorescence-conjugated antibodies: APC-CD11b (Clone: M1/70; eBioscience™), PE-Ly6G (Clone: 1A8; MilliporeSigma™), Fitc-CD4 (Clone: GK1.5; eBioscience™), APC-CD8 (Clone: 53–6.7; R&D Systems™), PE-Perforin (Clone: eBioOMAK-D; eBioscience™), PerCP-5.5-Granzyme (Clone: NGZB; eBioscience™), PerCP-5.5-Arginase (Polyclonal, Novus Biologicals™), and PE-iNOS (Clone: CXNFT, eBioscience™), PE-EpCAM (Clone: EBA-1, BD Biosciences). Cells were staining for 30 min at 4 °C and washed with PBS twice before examined. For intracellular protein markers (arginase, iNOS, granzyme, and perforin), cells were conducted with fixation/permeabilization procedure following the instruction (BD Biosciences, 555028) before being stained with fluorescently labeled antibodies for 30 min. Cells labeled with fluorescence-conjugated antibodies were then examined with BD LSR Fortessa Analyzer and analyzed with FlowJo v10.6.2. Prostate cancer cells labeled with PE-EpCAM were isolated with FACS Aria sorting system for following experiments.

### RNA sequence

Total RNA was extracted by Trizol (Invitrogen, Cata No. 15596026) following the manufacturer’s protocol. RNA quality was assessed by the RNA Nano 6000 Assay Kit of the Bioanalyzer 2100 system (Agilent Technologies, CA, USA). Degradation and contamination of RNA were checked on 1% agarose gels. RNA library construction was performed using TruSeq RNA Library Prep kit (Illumina). RNA was sequenced by HaploX (Jiangxi, China) in an Illumina platform.

### Real-time RT-PCR analyses

Total RNA was prepared by RNeasy Mini Kit (Invitrogen, Cata No. 15596026). RNA was used to synthesis cDNA using SuperScript III First-Strand Synthesis SuperMix (Invitrogen). The quantitative PCR was performed in triplicate using an ABI Prism7000 (Applied Biosystems) which was used to calculate the fold change by normalizing to Actin. The “2-ΔΔC(T)” method was applied to calculate the fold change by normalizing to GAPDH. All primers were designed with PrimerBank-MGH PG (https://pga.mgh.harvard.edu/primerbank/) and synthesized using Integrated DNA Technologies (Coralville, IA) (Additional file 1: Table S3).

### Statistics

All data are repeated at least 3 biological and technical triplicates. The data was representative of mean ± SD. Statistical differences were calculated by unpaired or paired Student’s *t*-test, two-sided log-rank test, one-way or two-way ANOVA, hypergeometric test, or Mann–Whitney *U* test using GraphPad Prism (GraphPad 5.0). *P* values of < 0.05 were considered significant. *P < 0.05, **P < 0.01, ***P < 0.001.

## Results

### CN133 has a high selectivity of class I HDAC target and can penetrate into the blood-prostate barrier effectively

HDAC inhibitor combination with immunotherapy can block cancers progression by improvement of immunogenicity through alter activation and function of macrophage and dendritic cells, decreasing the function of immunosuppressive effect in vivo and in vitro [12–14]. However, clinical trial with HDACi as single agent in the treatment of prostate cancer has produced disappointing results with high rates of side-effects [15]. Therefore, only few clinical researches were designed to test the anticancer efficacy of combination with immunotherapy in clinical trials in the prostate cancer. These mechanisms are likely multifactorial, and it may include low selectivity binding on class I HDACs and a limited penetration and distribution of HDACi in prostate cancer tumors because of existence of BPB [16–18], thus limiting effectiveness of HDACi in prostate cancer tumor.

**Fig. 1:**
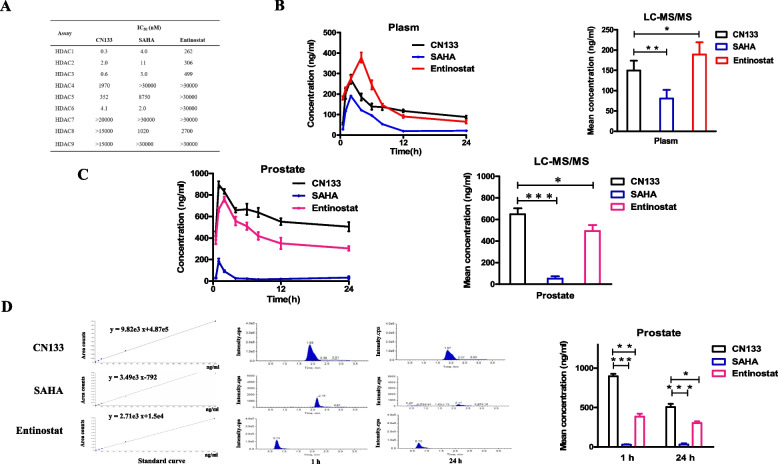
Compared to the classical HDAC inhibitors SAHA and entinostat, CN133 had higher HDAC 1, 2, and 3 enzyme selective inhibition and prostate tissue targeting. **A** In vitro enzyme inhibition activity experiments confirmed that the inhibitory efficiency of HDACs 1, 2, and 3 was significantly higher than that of SAHA and entinostat. **B** LC–MS/MS was applicated to detect drug concentrations in plasma and prostate tissues of mice at eight time points including 0.5 h, 1 h, 2 h, 4 h, 6 h, 8 h, 12 h, and 24 h. CN133 was found to be lower than entinostat in plasma on average but higher than SAHA in plasma on average. **C**, **D** Drug concentration of average value (**C**) and two time points (1 h and 24 h, respectively) (**D**) in the prostate tissue were significantly higher than entinostat and SAHA

Despite the challenges and difficulties in the drug discovery of therapeutics, the potential therapeutic benefits of HDAC inhibitors in prostate cancer tumor urged us to develop a novel higher BPB penetrant HDAC inhibitor. To accomplish this, we developed a new HDAC inhibitor CN133 and set out to test the inhibition of HDACs. As shown in Fig. 1A, when compared with SAHA and entinostat, CN133 exerted the most potent suppression on class I HDAC activity with smallest IC50 value of 0.3 nM, 2 nM, and 0.6 nM against HDACs 1, 2, and 3 (Fig. 1A). In order to verify BPB permeability of CN133, SAHA, and entinostat, we next carried out the in vivo blood and prostate tissue pharmacokinetic studies assay in the rats. HDACIs were administered intravenously to male rats (*n* = 40, each group) at 2 mg/kg, and the concentrations of HDACIs in the prostate and plasma were analyzed using LC–MS/MS at 8 time points. In vivo pharmacokinetic studies indicated that although CN133 had a lower mean concentration than entinostat in the plasm (Fig. 1B), CN133 displayed significant higher permeability property and a stability concentration in the prostate tissues than entinostat and SAHA (Fig. 1C, D). Overall, prostate pharmacokinetic results in vivo illustrated that CN133 effectively distributed into prostate tissue than FDA-approved HDAC inhibitor SAHA and entinostat.

### CN133 suppressed tumor progression by mechanisms of augmented immunity in the subcutaneous murine PCa mouse model

**Fig. 2:**
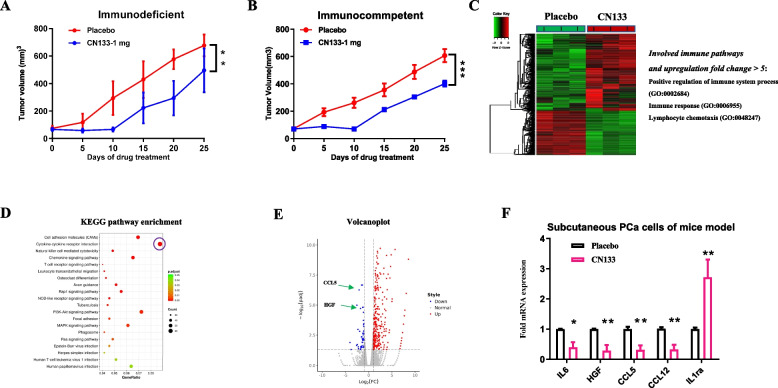
CN133 may improve anti-tumor effect by augment of immunity function. **A** Growth curve of RM1 prostate cancer subcutaneous tumors in immunodeficient BALB/c mice treated with placebo or low dose CN133 treatment. **B** Growth curve of RM1 prostate cancer subcutaneous tumors in immunocompetent C56BL/6 J mice treated with placebo or low dose CN133 treatment. **C** Microarray data from CN133 treatment (*n* = 3) and placebo tumor tissues (*n* = 3) in the xenograft C56BL/6 J mice were applied to identify differential genes between those two groups. **D** KEGG pathway enrichment analysis showed top 10 significantly different pathways in the CN133 treatment group relative to the placebo group. **E** Volcano plots showing different genes from RNA-seq data. **F** MDSC regulation cytokines were tested in the xenograft C56BL/6 J tumor tissues by RT-qPCR

To determine the effects of CN133 on prostate cancer in vivo, immunodeficient or immunocompetent mice were inoculated subcutaneously with RM1 cell line, respectively. Both tumor bearing mice with RM1 cells were randomized into two groups (*n* = 6, each group), respectively: placebo, CN133 (1 mg/kg). Although CN133 treatment decreased tumor growth in immunodeficient mice (26.8% growth inhibition; *p* < 0.01), treatment with CN133 resulted in more significant inhibition of tumor growth across the studied models of immunocompetent (36.5% growth inhibition; *p* < 0.001) (Fig. 2A, B). These data indicated that CN133 inhibited tumor progression through mechanisms that may be related to tumor immunity.

### CN133 may improve anti-tumor immunity by suppression of PMN-MDSC infiltrating and function in the subcutaneous PCa mouse model

Epigenetic therapy including HDAC inhibitors improved immunotherapeutic effect on tumors by increasing immunogenicity of cancer cells or remodeling the immunosuppressive tumor microenvironment [19]. To investigate whether CN133 could cause prostate cancer cells killing by inducing positive immune microenvironment, we conducted microarray analysis comparing the gene expression profile of CN133 treatment tumors and placebo treatment tumors in the allografts C57BL/J6 mice of immunocompetence (*n* = 3, each group). More than 1000 genes were identified that are differentially expressed between those two groups, including 351 significantly upregulated genes and 49 significantly downregulated genes (Fig. 2C). Gene expression analysis of RM1 allograft tumors revealed that CN133 induced a plethora of immune-related pathways (Fig. 2C). Meanwhile, KEGG pathways analysis showed a significant enrichment in cytokine-cytokine receptors pathway in the CN133 treatment group relative to the placebo group (Fig. 2D). Interesting, RNA-seq showed that CN133 treatment resulted in HGF and CCL5 downregulation significantly, the critical genes of accumulation of myeloid-derived suppressor cells (MDSC) (Fig. 2E) [20, 21]. To further validate the findings, we isolated the prostate cancer cells from C57BL/J6 mouse tumor tissues by fluorescence-activated cell sorting (FACS) and tested the expression of the Pro-MDSCs accumulation cytokines with RT-qPCR. Our data demonstrated that the lower levels of the Pro-MDSCs including IL6, CCL5, CCL12, and HGF were found in the CN133 treatment compared to the placebo group, and as an anti-MDSCs accumulation cytokine, IL-1ra was markedly upregulation in CN133 treatment compared to placebo group (Fig. 2F) [20, 21].

**Fig. 3:**
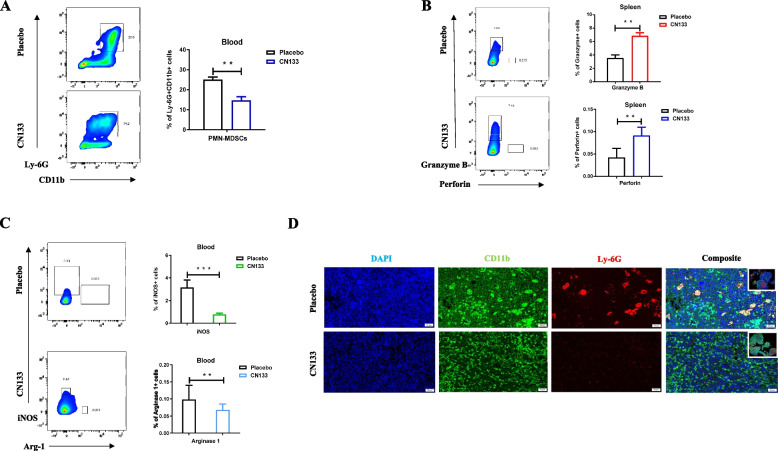
CN133 inhibit PCa growth in C56BL/6 J mice bearing murine RM1 subcutaneous tumors, mainly by downregulation of PMN-MDSC infiltration and function. **A** Flow cytometric analyses examined PMN-MDSC (CD11b + Ly-6G +) expression in the blood of subcutaneous PCa model in both of CN133 and placebo groups at day 25. **B**, **C** Flow cytometric analyses tested the expression of granzyme B and perforin (**B**), Arg-1, and iNOS (**C**) in the spleens of subcutaneous PCa model in the CN133 and placebo groups at day 25. **D** Representative fluorescent images of infiltration of PMN-MDSCs after day 25 of CN133 treatment or placebo treatment from the subcutaneous PCa tumor tissues; scale bar, 50 μm

Therefore, we examined the tumor infiltrating myeloid and lymphoid populations by flow cytometry and fluorescent images. Flow cytometric analyses of immune cells in the blood and spleen of tumors were statistically significant decreased in the PMN-MDSCs and mild upregulation of CD4 + and CD8 + T cells in the CN133 treatment group relative to the placebo treatment group (Fig. 3A, Additional file 2: Fig. S1A). However, markedly increased cytotoxic mediator secretion (granzyme B, perforin) in the CD8 + T cells (Fig. 3B) and reduced immunosuppressive enzymes secretion (Arg-1, iNOS) in the PMN-MDSCs were confirmed in the CN133 group by flow cytometric analyses (Fig. 3C). Similarly, microscope data showed that CN133 treatment in tumors was associated with slightly increased CD8 + TILs (Additional file 2: Fig. S1B) and significantly decreased infiltration of PMN-MDSCs compared to levels in placebo tumors (Fig. 3D).

This finding demonstrated that, although CN133 did not contribute to infiltration of CD8 + TILs, it markedly inhibited the number of PMN-MDSCs and secretion of immunosuppressive enzymes and promoted the positive cytotoxic mediator secretion from CD8 + T cells.

### CN133 enhanced the anticancer effects of PD-1 blockade in the subcutaneous prostate cancer allograft tumors

A common mechanism of immune evasion for prostate cancer is CD8 + T cell of dysfunction and poorly infiltration in tumors microenvironment [22]. The lack of cytotoxic effects of CD8 in prostate cancer may be dependent on high infiltration of immunosuppressive cells including regulatory T cells (Tregs) [23] and/or MDSCs [24]. Importantly, tumor cells can secret cytokines, including TGF-beta, IL-6, IL10, and VEGF, which commonly lead to the upregulation of PD-1/PD-L1 expression to evade immune detection [25]. Based on our data, we found there was significantly change in levels of cells of PMN-MDSCs and without change of the CD8 + TIL infiltration when CN133 alone was applied into our RM1 PCa model. Our animal data of CN133 treatment encouraged the combination of PD-1 blockade and HDAC inhibitor since PD-1 blockade can function by blocking the inhibitory interactions between tumor cells and CD8 + TILs [26].

**Fig. 4:**
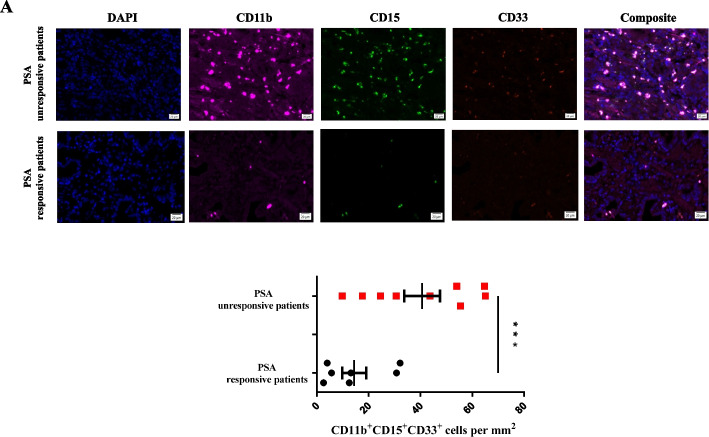
Unresponsive patients for PD-1 treatment had a significantly higher PMN-MDSC infiltration in TME when compared with responsive patients. **A** Tumor-infiltrating PMN-MDSC cell lineages (CD11b + CD15 + CD33 +) in PD-1-treated PCa samples; scale bar, 20 μm

**Fig. 5:**
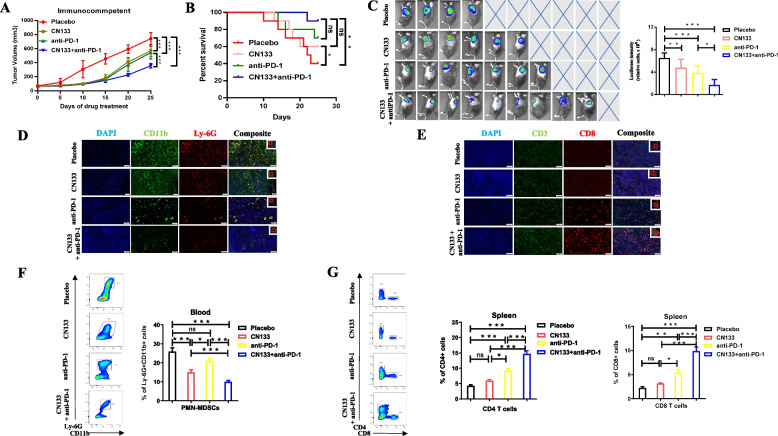
CN133 enhanced the anticancer effects of PD-1 blockade in C56BL/6 J mice of RM1 subcutaneous tumors by inhibited PMN-MDSC infiltration and enhanced CD8 T cell infiltration in TME. **A** Tumor volume of RM1 prostate cancer subcutaneous tumors treated with placebo, CN133, anti-PD-1 (1 mg/kg), or CN133 (1 mg/kg) combined with anti-PD-1. **B** Survival percentage were assessed after treatment of placebo, CN133, anti-PD-1, or CN133 plus anti-PD-1 in C57BL/J6 models. **C** Bioluminescent images (BLI) showing the luciferase intensity of subcutaneous tumors after treatments of placebo, CN133, anti-PD-1, or CN133 plus anti-PD-1. **D** In situ analyses of PMN-MDSC population of tumor by immunofluorescence staining in the TME of placebo, CN133, anti-PD-1, or CN133 plus PD-1 treatment C57BL/J6 mice; scale bar, 50 μm. **E** Immunofluorescent images showed the infiltration of CD3 + CD8 + T cells on C57BL/J6 subcutaneous PCa tumor tissues; scale bar, 50 μm. **F**, **G** Flow cytometric analyses showing percentages numbers of the PMN-MDSCs (**F**) and CD4 and CD8 T cells (**G**) in the spleens of placebo, CN133, anti-PD-1, or CN133 plus anti-PD-1 treatment of C57BL/J6 PCa mice

Firstly, we collected the PCa patient samples of anti-PD-1 treatment in our hospital. Across our cohorts, 7 patients had achieved an PSA response and 9 did not after anti-PD-1 treatment. We evaluated post-treatment tumor infiltrating PMN-MDSC cell in both group and found a significantly higher PMN-MDSC numbers in the PSA unresponsive group as compared to the PSA responsive group (Fig. 4A). Furthermore, to test whether PD-1 blockade and HDAC inhibitors combination potentiates an enhanced immune response, we employed a combination therapy model system. The tumors responded to the anti–PD-1 plus CN133 treatment remarkably well than single treatment (CN133 or anti-PD-1) or placebo, which showed the most effective inhibition of primary tumors 25 days after combinational treatment initiation after allografts using RM1 PCa cell line (Fig. 5A and 5C) or MyC-CaP PCa cell line (Additional file 2: Fig. S2A and S2C) in C57BL/J6 and FVB mouse models, respectively. Meanwhile, the combination of anti–PD-1 antibody and CN133 increased the survival compared to the CN133 or anti-PD-1 single treatment in both mice models (Fig. 5B, Additional file 2: Fig. S2B). Furthermore, we examined the tumor infiltrating CD8 + TILs and PMN-MDSC population by microscope and found that combination treatment of CN133 and anti-PD-1 antibody has been shown to alter the tumor microenvironment not only by reducing PMN-MDSC cell population but also significantly enhancing CD8 + TIL infiltration, comparing with CN133 or anti-PD-1 single treatment group in C57BL/J6 xenografts mice model (Fig. 5D, E). Additionally, blood and spleen samples were also collected from tumor-bearing mice and subjected to flow cytometric analysis to determine the levels of immune cell infiltration and enzymes expression. The combination group also shown a significant reduction in PMN-MDSC levels as compared to the placebo group or any single treatment group (Fig. 5F). As expected, combination treatment had an increased CD4 + and CD8 + T cells presence in the spleens when compared with the placebo group, CN133, or anti-PD-1 treatment alone (Fig. 5G). Combination treatment significantly reduced enzymes release from the PMN-MDSCs (Arg-1, iNOS) as compared with anti-PD-1 alone or placebo (Additional file 2: Fig. S2D). Meanwhile, compared to anti-PD-1 alone or placebo, remarkably increased cytotoxic mediator secretion (granzyme B, perforin) in the splenic CD8 + T cells was confirmed in the combination group by flow cytometric analyses, when compared with the CN133 and control groups (Additional file 2: Fig. S2E). Taken together, our data suggest that PD-1 blockade immunotherapy combined with CN133 could significantly reduce the immune escape of the tumor via increased CD8 + TILs and decreased presence of PMN-MDSC population.

### Combined CN133 with PD-1 blockade could not show positively synergistic antitumor efficacy on bone metastatic PCa mouse model

Bone is the most frequent metastatic place for prostate cancer, and 70% of patients endure the skeletal impairment in the metastatic castration-resistant prostate cancer (mCRPC) period [27]. Moreover, bone metastases represent a high disease-specific mortality in mCRPC [28]. It has been proven that targeting CTLA4 or PD-1/PD-L1 had poorer response in mCRPC patients with bone metastasizes compared with those without bone metastasize [3, 29]. Our results showed that treatment with CN133 combination with anti-PD-1 could improve the response of subcutaneous tumors to anti-PD-1 treatment by activating the immune system. PMN-MDSCs which were originated from immature myeloid cells of the bone marrow could migrate to TME to promote resistance to CD8 + cells and enhance the immune escape of the tumor via production of immunosuppressive enzymes such as Arg-1 and iNOS. We wonder whether the anti-tumor ability of combination therapy using CN133 and anti-PD-1 co-treatment could be effective against bone metastasis of prostate cancer.

**Fig. 6:**
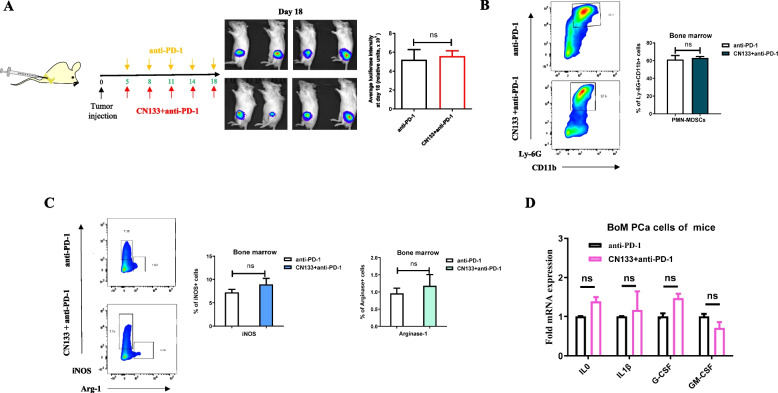
Combined CN133 with PD-1 did not improve the anti-tumor efficacy on the FVB mice bearing murine RM1 bone metastatic PCa tumors. **A** Timeline of mouse models of bone metastasis PCa with PD-1 or PD-1 plus CN133 treatment schedules (left) and BLI showing the luciferase intensity of bone tumors at day 25 (right). **B**, **C** Flow cytometric analyses examined positive percentages of MDSCs (**B**) and Arg-1 and iNOS (**C**) in the bone marrow PCa at day 25. **D** Activity cytokines of MDSCs were tested in the RM1 cell of bone metastatic PCa tumor by RT-qPCR

We therefore devised and evaluated the anti-tumor effects of CN133 combination anti-PD-1 in an organ-specific the microenvironment of bone metastasizes prostate cancer using mouse model of RM1. We found that anti-PD-1 combination with CN133 treatment did not show the same effective with the subcutaneous mouse model of RM1. Combination treatment did not reduce tumor volume in the bone marrow compared with single-agent PD-1 treatment (Fig. 6A, Additional file 2: Fig. S3A). Compared to anti-PD-1 treatment, immune cells in bone metastasis tumors also did not show significantly downregulation of the PMN-MDSCs or upregulation of CD8 + TILs in the combination treatment in the TME from flow cytometric results (Fig. 6B, Additional file 2: Fig. S3B). However, the infiltration of CD4 + T cells in the bone marrow was increased significantly (Additional file 2: Fig. S3B). Meanwhile, secretion changes of Arg-1, iNOS, granzyme, and perforin by PMN-MDSCs and CD8 + TILs were not observed in CN133 combination with anti-PD-1 by flow cytometric data when compared with anti-PD-1 treatment (Fig. 6C, Additional file 2: Fig. S3C). Combination of CN133 and anti-PD-1 could not improve anti-tumor effectiveness. It demonstrated that a discrepancy immunological niche in the bone microenvironment compared with soft tumor tissues.

To exam the effect of CN133 on the level of the bone metastases prostate cancer, we isolated and analyzed the prostate cancer cells from bone metastases RM1 model by FACS. We focus on gene expression which was associated with PMN-MDSC activation to determine which of the modulated transcripts were modulated by CN133 [21]. Within this pool, in RM1 cells, we did not observe a significant change that associated with activity cytokines of PMN-MDSCs in the CN133 plus anti-PD-1 group relative to the anti-PD-1 alone including G-CSF, IL-1β, GM-CSF, and IL10 (Fig. 6D).

Therefore, our data suggest that combination of CN133 and anti-PD-1 did not show synergistic efficacy of anti-tumor in osseous PCa, possibly owing to limited efficacy on the secretion cytokines of PMN-MDSC activation from PCa cells.

## Discussion

Prostate cancer has remained relatively resistance to ICB which was correlated with the lack of intratumoral T cells [30, 31]. HDACIs improved immunogenicity through enhancing the cancer-germline antigen expression or improving the cytotoxic T cell populations in TME [32–34]. In this research, we tested our new HDACi, CN133, to characterize the anti-tumor potency through immunologic regulation by a series of in vivo experiments. Firstly, we found that the anti-tumor activity of CN133 in immunocompetent mouse was more significant than in immunodeficient mouse on subcutaneous allograft model of prostate cancer. Moreover, in vivo analyses demonstrated that these changes were immunologically relevant, as CN133 alone treatment resulted in remarkably decreased PMN-MDSC population and functional molecules secretion of PMN-MDSCs in the TME and blood, and improved CD8 + T cells mediated lysis to prostate cancer cells by secretion of granzyme B and perforin. Furthermore, our data in vivo showed that CN133 combination with anti-PD-1 treatment can enhance antitumor effect by improved CD8 + T cell infiltration and upregulate the secretion of effector enzymes (granzyme B, perforin) in the TME. Mechanistically, the trend towards increased anti-tumor activity of CN133 treatment may correlate with downregulation of the PMN-MDSC recruitment by reduction of the secretion of cytokines from PCa cells. However, combination of CN133 and anti-PD-1 was not significantly synergistic efficacy when prostate cancer cells were metastases to bone in the preclinical mouse model. This may in part be explained by the poor effect of combination therapy on the secretion chemokines of PMN-MDSC activation from PCa cells.

HDAC inhibitors have been in clinical development as a single agent for hematopoietic malignancies such as myelodysplasia, a precursor of leukemia, and chronic myelomonocytic leukemia [12]. However, it has low effectivity with alone treatment of HDACi in solid tumor patients from clinical trial results [15]. Recently, novel combinations of epigenetic drugs in human clinical trials, especially for HDACIs, combined with immunotherapy have shown positively synergy efficacy in multiple solid carcinomas by regulating the cytokine release to activate innate immune cell infiltration in the tumor microenvironment [35–37]. Although HDACIs may play a suppressive function in vitro, it was not approved as a first-line treatment owing to a high side-effect in prostate cancer clinical trials [15]. HDACIs with high binding affinity and selectivity of class I HDACs and high ability to cross the BPB can be the key approaches to improve therapeutic benefits because of the serious side-effect in clinical trials and existence of blood barrier in prostate tissue. We developed a new HDAC inhibitor, CN133, which has been approved in performing clinical trials as the PET imaging tracer in brain disorders such as Alzheimer’s disease and schizophrenia [10, 11], which presented a higher class I HDAC binding affinity and selectivity in this study when compared to classic HDACIs SAHA and entinostat. We tested the BPB permeability of CN133 and found that in prostate tissues, it also showed a significantly higher accumulation set of CN133 than SAHA and entinostat. These physicochemical properties of CN133 support therapeutic potency for the treatment of prostate cancer.

In our previous study, we found CN133 could inhibit the proliferation of prostate cancer cell line in vitro [38]. Furthermore, we designed experiments to assess the ability of anti-prostate cancer of CN133 by building allograft models of prostate cancer using immunodeficient or immunocompetent mice. Those data analyses demonstrated that low-dose CN133 (1 mg/kg) decreased tumors volume more significantly in immunocompetent mice relative to immunodeficient mice. It has previously been reported that low-dose epigenetic therapy resulted in a reversal of the immunosuppressive microenvironment to suppress the tumor growth [39, 40]. Interestingly, we also found that compared to high-dose CN133 (5 mg/kg), CN133 with low dose (1 mg/kg) conferred to a marked reduction in tumors volume in xenograft prostate cancer model of nude mice in our previous study [38]. Although nude mice are useful model to assess tumor xenograft development because of athymic congenitally, extrathymic T cells including CD4 + or CD8 + cells could partially develop maturation as age growth in nude mice [41, 42]. Therefore, data from this time and our previous results demonstrated that low-dose CN133 may efficaciously suppress prostate cancer growth by augment of immunogenicity.

Tumors always induced immune evasion mainly by improving immunosuppressive cells activity or reducing cytotoxic T cell infiltration or activity. CD8 + TILs, as the major antitumoral immune cells, are always induced to exhaust or disfunction mainly by tumor cells through upregulation of PD-1/PD-L1 or recruitment of immunosuppressive myeloid cells. MDSCs which were the key cells in the immunosuppressive function were well known inhibitors of T cell infiltration and activity by secretion of immunosuppressive enzymes. In circulation or prostate cancer tissues, MDSC population were correlated with tumor progress from localized to metastatic disease [43, 44]. HDAC inhibitors have been proven to improve immunotherapeutic effect on tumors by deletion of immunosuppressive MDSCs [45]. In this study, we found that it was significantly decreased in the PMN-MDSCs with marked reduction of immunosuppressive enzymes secretion (Arg-1, iNOS) and mild upregulation of CD8 + TILs with significantly elevated cytotoxic mediator secretion (granzyme B, perforin) in the immunocompetent mice on CN133 treatment.

Based on those results, we hypothesize that combination ICB with CN133 may be a potential therapeutic option for prostate cancer because of a poor reaction for ICB therapy alone [8, 9]. Our data represented that combined anti-PD-1 with CN133 showed good therapeutic effects in subcutaneous prostate cancer, but not in bone metastatic prostate cancer, indicating different immunological niches in soft tumor tissue and bone marrow. Actually, from preclinical research to clinical data, combination HDAC inhibitor with ICB immunotherapy can overcome resistance to ICB treatment alone including breast and pancreatic cancers, metastatic uveal melanoma, recurrent or metastatic squamous cell carcinomas of the head and neck, and metastatic non-small cell lung cancer [5, 7–9]. However, there was not any data about combination HDACIs with ICBs in the preclinical or clinical research of PCa. As far as we know, this is the first study about the effect of HDAC inhibitor combined with anti-PD-1 treatment on PCa.

Although anti-PD-1/PD-L1 monotherapies, even combination anti-PD-1/PD-L1 with cytotoxic T lymphocyte antigen 4 (CTLA-4) to maximize the blockade of checkpoint pathways can increase tumor-infiltrating T cells in the prostate, it always showed limited benefits in patients with prostate cancer in clinical trials [4, 46]. Many mechanisms involved tumor immune escape by blocking the immune checkpoint blockade (ICB) such as PD-1/PD-L1 in prostate cancer [47, 48]. A lower tumor mutational load, which made less antigens from mutation, leads a fewer benefit from ICB therapy. That is the reason patients with microsatellite instability (MSI) showed an impressive response rate than without MSI patients in clinical trials [49]. Another important reason for this phenomenon is that tumors produce cytokines to suppress the immune checkpoint blockade therapy by the recruitment of immunosuppressive cells [50]. Another factor that correlated with the sensitivity to anti-PD-1 treatment is neutrophil-to-lymphocyte ratio [51, 52]. Our preclinical data of subcutaneous PCa model is consistent with clinical results showing that anti-PD-1 treatment alone can lead to increased CD8 + T cell infiltration and activation of positive immune molecules such as perforin but failed to affect PMN-MDSC infiltration significantly in the TME relative to placebo treatment. Furthermore, our data showed that CN133 in combination with anti-PD-1 improved efficacy significantly which was achieved in the subcutaneous murine PCa models by decreasing tumor-infiltrating PMN-MDSCs and their expression of functional molecules Arg-1 and iNOS. Of relevance to our study, previous evidences both in preclinical and clinical studies have proved that combining ICB with MDSC-targeted therapies using cabozantinib showed promising antitumor activity in metastatic castration-resistant prostate cancer (mCRPC) [20, 53]. Although CN133 combination with anti-PD-1 did not reduce the PMN-MDSC population in the murine models of bone metastases, it could suppress the infiltration of PMN-MDSCs in the TME of soft tissue PCa. Our data demonstrating the impact of CN133 on PMN-MDSCs also explored the possibility that combination CN133 with ICB in the treatment of metastatic castration-resistant prostate cancer (mCRPC) patients in clinical.

However, further assessing the murine models of bone metastases to test the efficacy of combination therapy, we found it is insufficient to generate antitumor responses compared to anti-PD-1 alone. Previous clinical research showed that ICB monotherapies or combination regimens targeting ICB showed poorer clinical benefit in patients with bone metastases than without bone metastases [3, 4]. A recent study suggested that the presence of osseous prostate cancer cells limited the effect of immune checkpoint therapy by favoring the development of Th17 and restraining Th1 lineage development in the bone marrow [54]. In addition, another research demonstrated that tumor-specific myeloid cells in bone metastasis microenvironment can produce local chemokine CCL20 to increase the exhaustion of T cell population [55].

Interestingly, our preliminary data suggest that PMN-MDSC populations as the myeloid subpopulations producing within the bone marrow were significantly more than the subcutaneous TME of soft tissue PCa and could not be restrained by PD-1 blocking therapy in the context of bone metastatic PCa. This may in part be explained by that the bone marrow is the origin of immunocyte and induced a more activated immunosuppressive microenvironment by osseous prostate cancer cells easily. Meanwhile, our data presented here suggested that CN133 plus anti-PD-1 fail to suppress the key cytokine production by prostate cancer cells of the bone marrow involved in the activation of MDSCs, which might explain why combination therapy played a limited effect in our murine PCa models of bone metastases. Our data strongly indicates that the drug which can block the cytokines of MDSC activation secreted by osseous PCa cells might show a positively synergistic efficacy for the effects of ICB on bone metastatic PCa.

## Conclusions

Our data supported that the combination CN133 with anti-PD-1 can circumvent the resistance to anti-PD-1 treatment by expansion of CD8 + T cells and inhibition of PMN-MDSCs in the soft tissue TME of subcutaneous PCa model, but not showing significantly anti-tumor activity in bone metastatic PCa. The preclinical study presented here will provide rational immunotherapy strategies of combination CN133 with ICB to expand the efficacy in soft tissue prostate cancer. Furthermore, these data suggest that resistance to ICB in bone metastases might be overcome by simultaneously blocking the cytokines of MDSC activation secreted by osseous PCa cells.

### Supplementary Information

**Additional file 1: Table S1.** Animals are grouped according to a random number provided by the supplier. **Table S2.** Baseline demographic and clinical characteristics of anti-PD1 treatment patients. **Table S3.** Primers for quantitative RT-PCR.

**Additional file 2: Fig. S1.** CN133 did not change the infiltration of CD4 and CD8 T cells in RM1 subcutaneous tumors. A. Flow cytometric data showing resulting of CD4+ and CD8+ T cells in spleens of CN133 and placebo groups at day 25. B. Fluorescent images of CD3+CD8+ T cells in placebo and CN133 treatment of RM1 subcutaneous tumors from C56BL/6J mice. Scale bar, 50 μm. **Fig. S2.** Combination of CN133 with anti-PD-1 inhibit PCa growth and improve the survival percentage in FVB mice bearing MyC-CaP subcutaneous tumors. Synergistic effect of CN133 with anti-PD-1 enhanced CD8 T+ cell population and function, reduced the secretion of Arg-1 and iNOS in C57BL/J6 models. A. Xenografts tumor volume of FVB mice with MyC-CaP prostate cancer cell line treated with placebo, CN133, anti-PD-1 (1mg/kg) or CN133 (1mg/kg) combined with anti-PD-1. B. Survival percentage of FVB subcutaneous PCa mice models were assessed after treatment of placebo, CN133, anti-PD-1 or CN133 plus anti-PD-1. C. BLI intensity of subcutaneous tumors of FVB mice after treatments of Placebo, CN133, anti-PD-1 or CN133 plus anti-PD-1. D and E. Flow cytometric analyses examined positive percentages of Arg-1 and iNOS in the PMN-MDSCs (D), and granzyme B and perforin in the CD8+ T cells (E) in spleens of placebo, CN133, anti-PD-1 or CN133 plus anti-PD-1, treatment of subcutaneous C57BL/J6 PCa mice. **Fig. S3.** CN133 combination of PD-1 did not improve CD8 T cell population and function on the FVB mice bearing murine RM1 bone metastatic PCa tumors. A. MRI images of tumor-bearing bones in mice treated with PD-1 or PD-1 plus CN133 at day 25. B and C. CD4 and CD8 T cells (B), and granzyme B and perforin effector cytokine (C) were quantified by flow cytometric analyses in the bone marrow of this PCa mice models at day 25.

## Data Availability

The data that support the findings of this study are available on request from the corresponding author upon reasonable request.

## Abbreviations

Arg-1: Arginase-1
BLI: Bioluminescence imaging
BPB: Blood-prostate barrier
CTLA-4: Cytotoxic T lymphocyte antigen 4
FACS: Fluorescence-activated cell sorting
HDACIs: HDAC inhibitors
ICB: Immune checkpoint blockade
iNOS: Inducible nitric oxide synthase
mCRPC: Metastatic castration-resistant prostate cancer
MDSC: Myeloid-derived suppressor cells
PCa: Prostate cancer
PMN-MDSCs: Polymorphonuclear myeloid-derived suppressor cells
PSA: Prostate-specific antigen
TME: Tumor microenvironment
