# Supplementary material for: A new histone deacetylase inhibitor remodels the tumor microenvironment by deletion of polymorphonuclear myeloid-derived suppressor cells and sensitizes prostate cancer to immunotherapy
Source: BMC Med. 2023 Oct 25;21:402. doi: 10.1186/s12916-023-03094-0 (PMC10601128; doi:10.1186/s12916-023-03094-0)
Supplement: Supplementary file 1 — Additional file 1: Table S1. Animals are grouped according to a random number provided by the supplier. Table S2. Baseline demographic and clinical characteristics of anti-PD1 treatment patients. Table S3. Primers for quantitative RT-PCR. [file 12916_2023_3094_MOESM1_ESM.zip › additional file 1/Table S2R4.docx]

**Table S2. Baseline demographic and clinical characteristics of anti-PD1 treatment patients**

| **PSA Unresponsive** | **Total (9)** |  | **PSA Unresponsive** | **Total (7)** |
| --- | --- | --- | --- | --- |
| **Age** |  |  | **Age** |  |
| <70 | 4 |  | <70 | 2 |
| ≥70 | 5 |  | ≥70 | 5 |
| **BMI** |  |  | **BMI** |  |
| ＜25 | 5 |  | ＜25 | 4 |
| ≥25 | 4 |  | ≥25 | 3 |
| **PSA（ng/ml）** | |  | **PSA（ng/ml）** |  |
| <10 | 0 |  | <10 | 0 |
| 10~20 | 2 |  | 10~20 | 1 |
| >20 | 7 |  | >20 | 6 |
| **Gleason** |  |  | **Gleason** |  |
| ≤6 | 0 |  | ≤6 | 0 |
| 7 | 2 |  | 7 | 3 |
| ≥8 | 7 |  | ≥8 | 4 |
| **T stage** |  |  | **T stage** |  |
| ≤T2a | - |  | ≤T2a | - |
| T2b | - |  | T2b | - |
| ≥T2c | 9 |  | ≥T2c | 7 |
| **Clinical stage** |  |  | **Clinical stage** |  |
| Ⅰ | - |  | Ⅰ | - |
| Ⅱ | - |  | Ⅱ | - |
| Ⅲ | - |  | Ⅲ | - |
| Ⅳ | 9 |  | Ⅳ | 7 |
|  |  |  |  |  |
