# Supplementary material for: A new histone deacetylase inhibitor remodels the tumor microenvironment by deletion of polymorphonuclear myeloid-derived suppressor cells and sensitizes prostate cancer to immunotherapy
Source: BMC Med. 2023 Oct 25;21:402. doi: 10.1186/s12916-023-03094-0 (PMC10601128; doi:10.1186/s12916-023-03094-0)
Supplement: Supplementary file 1 — Additional file 1: Table S1. Animals are grouped according to a random number provided by the supplier. Table S2. Baseline demographic and clinical characteristics of anti-PD1 treatment patients. Table S3. Primers for quantitative RT-PCR. [file 12916_2023_3094_MOESM1_ESM.zip › additional file 1/Table S3R4.docx]

**Table S3. Primers for quantitative RT-PCR**

| **Gene Forward Reverse** | | |
| --- | --- | --- |
| IL6  CCL5  CCL12  HGF  IL-1ra   1. CSF   IL-1β  GM-CSF  IL10 | TCTATACCACTTCACAAGTCGGA  TTTGCCTACCTCTCCCTCG  ATTTCCACACTTCTATGCCTCCT  ACTTCTGCCGGTCCTGTTG  TAGACATGGTGCCTATTGACCT  GCACTATGGTCAGGACGAGAG  TTCAGGCAGGCAGTATCACTC  GGCCTTGGAAGCATGTAGAGG  GCTGGACAACATACTGCTAACC | GAATTGCCATTGCACAACTCTTT  CGACTGCAAGATTGGAGCACT  ATCCAGTATGGTCCTGAAGATCA  CCCCTGTTCCTGATACACCT  TCGTGACTATAAGGGGCTCTTC  GGGGAAATACCCGATAGAGCC  GAAGGTCCACGGGAAAGACAC  GGAGAACTCGTTAGAGACGACTT  ATTTCCGATAAGGCTTGGCAA |
