# Supplementary figures and images for: A new histone deacetylase inhibitor remodels the tumor microenvironment by deletion of polymorphonuclear myeloid-derived suppressor cells and sensitizes prostate cancer to immunotherapy
Source: BMC Med. 2023 Oct 25;21:402. doi: 10.1186/s12916-023-03094-0 (PMC10601128; doi:10.1186/s12916-023-03094-0)

**A**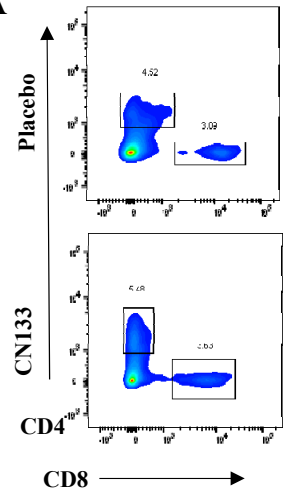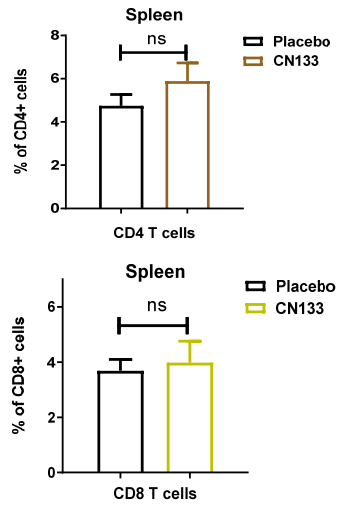**B**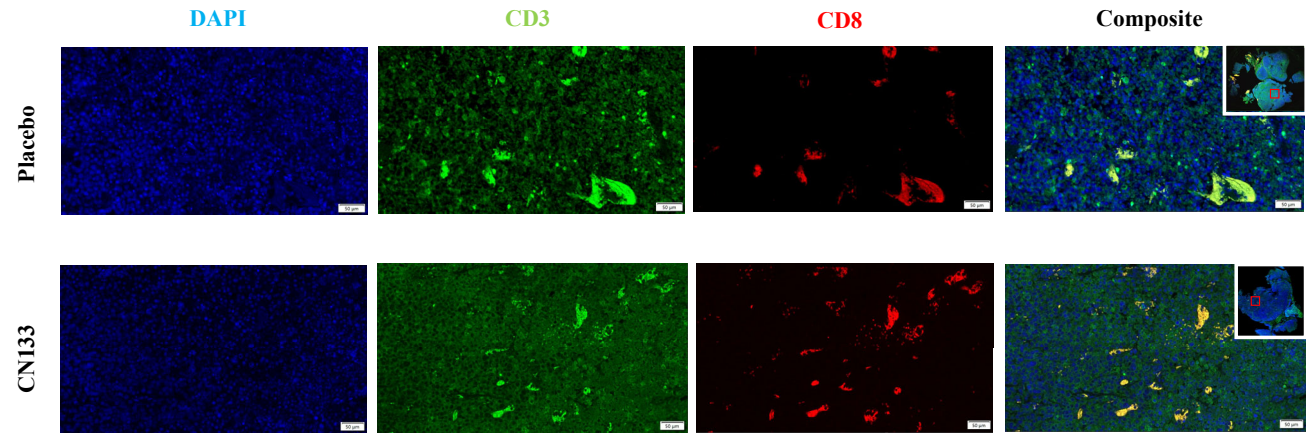

Supplement: Supplementary file 2 — Additional file 2: Fig. S1. CN133 did not change the infiltration of CD4 and CD8 T cells in RM1 subcutaneous tumors. A. Flow cytometric data showing resulting of CD4+ and CD8+ T cells in spleens of CN133 and placebo groups at day 25. B. Fluorescent images of CD3+CD8+ T cells in placebo and CN133 treatment of RM1 subcutaneous tumors from C56BL/6J mice. Scale bar, 50 μm. Fig. S2. Combination of CN133 with anti-PD-1 inhibit PCa growth and improve the survival percentage in FVB mice bearing MyC-CaP subcutaneous tumors. Synergistic effect of CN133 with anti-PD-1 enhanced CD8 T+ cell population and function, reduced the secretion of Arg-1 and iNOS in C57BL/J6 models. A. Xenografts tumor volume of FVB mice with MyC-CaP prostate cancer cell line treated with placebo, CN133, anti-PD-1 (1mg/kg) or CN133 (1mg/kg) combined with anti-PD-1. B. Survival percentage of FVB subcutaneous PCa mice models were assessed after treatment of placebo, CN133, anti-PD-1 or CN133 plus anti-PD-1. C. BLI intensity of subcutaneous tumors of FVB mice after treatments of Placebo, CN133, anti-PD-1 or CN133 plus anti-PD-1. D and E. Flow cytometric analyses examined positive percentages of Arg-1 and iNOS in the PMN-MDSCs (D), and granzyme B and perforin in the CD8+ T cells (E) in spleens of placebo, CN133, anti-PD-1 or CN133 plus anti-PD-1, treatment of subcutaneous C57BL/J6 PCa mice. Fig. S3. CN133 combination of PD-1 did not improve CD8 T cell population and function on the FVB mice bearing murine RM1 bone metastatic PCa tumors. A. MRI images of tumor-bearing bones in mice treated with PD-1 or PD-1 plus CN133 at day 25. B and C. CD4 and CD8 T cells (B), and granzyme B and perforin effector cytokine (C) were quantified by flow cytometric analyses in the bone marrow of this PCa mice models at day 25. [file 12916_2023_3094_MOESM2_ESM.zip › additional file 2/Fig.S1R4.pdf]

**A**

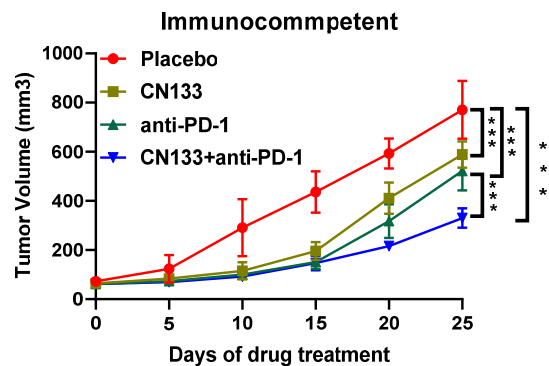

**B**

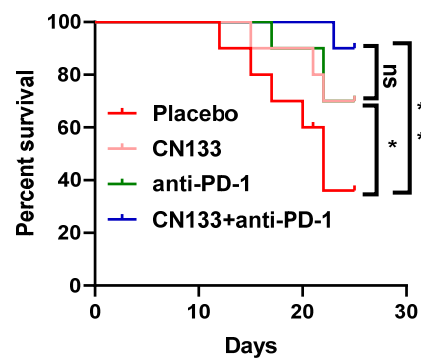

**C**

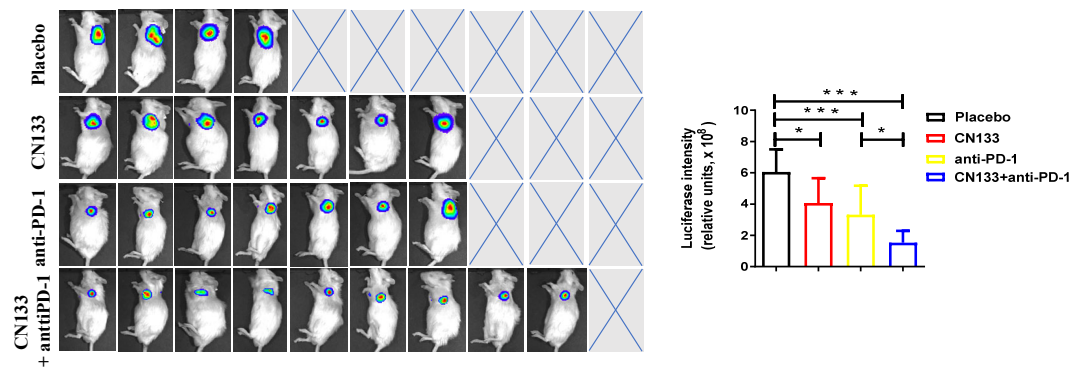

**D**

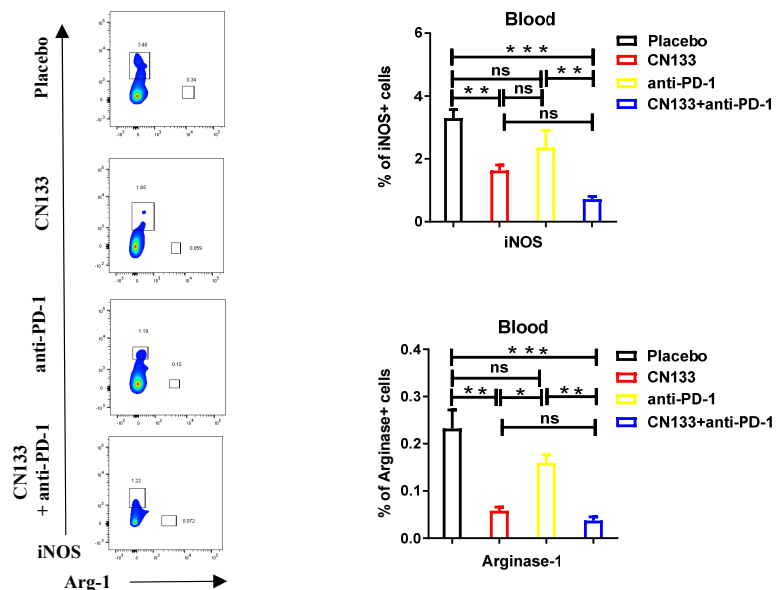

**E**

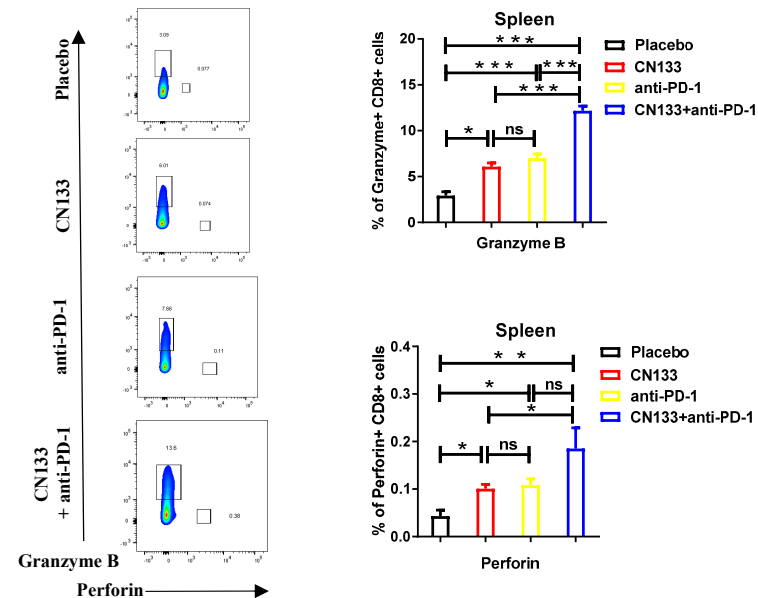

Supplement: Supplementary file 2 — Additional file 2: Fig. S1. CN133 did not change the infiltration of CD4 and CD8 T cells in RM1 subcutaneous tumors. A. Flow cytometric data showing resulting of CD4+ and CD8+ T cells in spleens of CN133 and placebo groups at day 25. B. Fluorescent images of CD3+CD8+ T cells in placebo and CN133 treatment of RM1 subcutaneous tumors from C56BL/6J mice. Scale bar, 50 μm. Fig. S2. Combination of CN133 with anti-PD-1 inhibit PCa growth and improve the survival percentage in FVB mice bearing MyC-CaP subcutaneous tumors. Synergistic effect of CN133 with anti-PD-1 enhanced CD8 T+ cell population and function, reduced the secretion of Arg-1 and iNOS in C57BL/J6 models. A. Xenografts tumor volume of FVB mice with MyC-CaP prostate cancer cell line treated with placebo, CN133, anti-PD-1 (1mg/kg) or CN133 (1mg/kg) combined with anti-PD-1. B. Survival percentage of FVB subcutaneous PCa mice models were assessed after treatment of placebo, CN133, anti-PD-1 or CN133 plus anti-PD-1. C. BLI intensity of subcutaneous tumors of FVB mice after treatments of Placebo, CN133, anti-PD-1 or CN133 plus anti-PD-1. D and E. Flow cytometric analyses examined positive percentages of Arg-1 and iNOS in the PMN-MDSCs (D), and granzyme B and perforin in the CD8+ T cells (E) in spleens of placebo, CN133, anti-PD-1 or CN133 plus anti-PD-1, treatment of subcutaneous C57BL/J6 PCa mice. Fig. S3. CN133 combination of PD-1 did not improve CD8 T cell population and function on the FVB mice bearing murine RM1 bone metastatic PCa tumors. A. MRI images of tumor-bearing bones in mice treated with PD-1 or PD-1 plus CN133 at day 25. B and C. CD4 and CD8 T cells (B), and granzyme B and perforin effector cytokine (C) were quantified by flow cytometric analyses in the bone marrow of this PCa mice models at day 25. [file 12916_2023_3094_MOESM2_ESM.zip › additional file 2/Fig.S2R4.pdf]

A

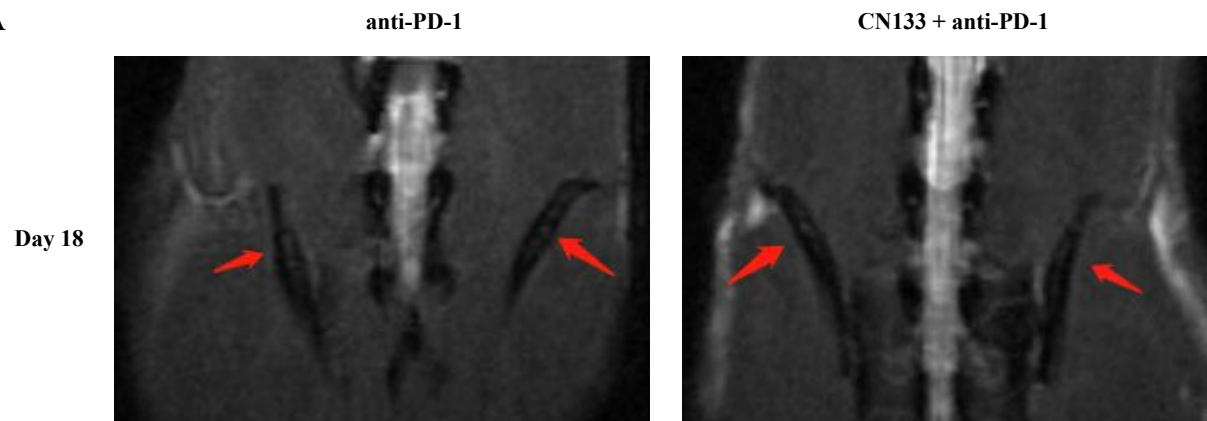

B

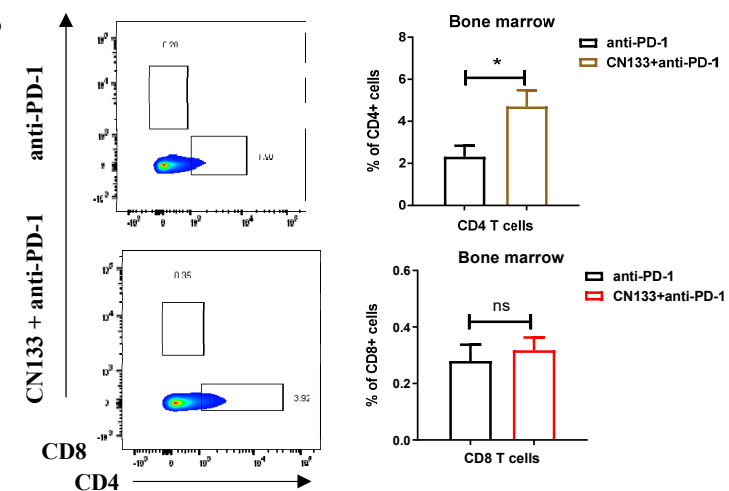

C

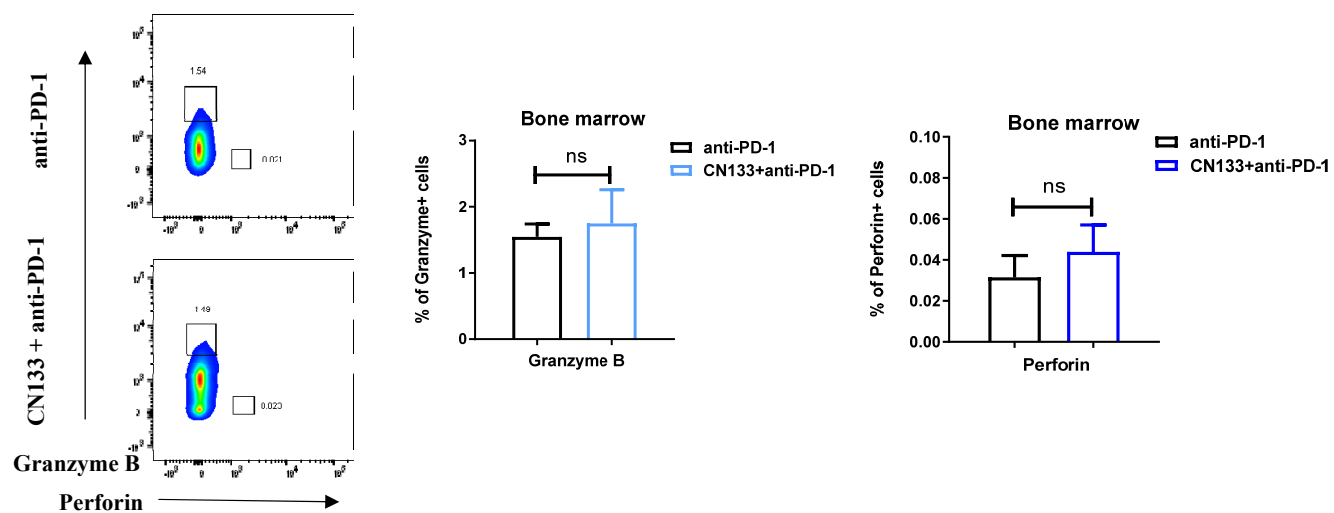

Supplement: Supplementary file 2 — Additional file 2: Fig. S1. CN133 did not change the infiltration of CD4 and CD8 T cells in RM1 subcutaneous tumors. A. Flow cytometric data showing resulting of CD4+ and CD8+ T cells in spleens of CN133 and placebo groups at day 25. B. Fluorescent images of CD3+CD8+ T cells in placebo and CN133 treatment of RM1 subcutaneous tumors from C56BL/6J mice. Scale bar, 50 μm. Fig. S2. Combination of CN133 with anti-PD-1 inhibit PCa growth and improve the survival percentage in FVB mice bearing MyC-CaP subcutaneous tumors. Synergistic effect of CN133 with anti-PD-1 enhanced CD8 T+ cell population and function, reduced the secretion of Arg-1 and iNOS in C57BL/J6 models. A. Xenografts tumor volume of FVB mice with MyC-CaP prostate cancer cell line treated with placebo, CN133, anti-PD-1 (1mg/kg) or CN133 (1mg/kg) combined with anti-PD-1. B. Survival percentage of FVB subcutaneous PCa mice models were assessed after treatment of placebo, CN133, anti-PD-1 or CN133 plus anti-PD-1. C. BLI intensity of subcutaneous tumors of FVB mice after treatments of Placebo, CN133, anti-PD-1 or CN133 plus anti-PD-1. D and E. Flow cytometric analyses examined positive percentages of Arg-1 and iNOS in the PMN-MDSCs (D), and granzyme B and perforin in the CD8+ T cells (E) in spleens of placebo, CN133, anti-PD-1 or CN133 plus anti-PD-1, treatment of subcutaneous C57BL/J6 PCa mice. Fig. S3. CN133 combination of PD-1 did not improve CD8 T cell population and function on the FVB mice bearing murine RM1 bone metastatic PCa tumors. A. MRI images of tumor-bearing bones in mice treated with PD-1 or PD-1 plus CN133 at day 25. B and C. CD4 and CD8 T cells (B), and granzyme B and perforin effector cytokine (C) were quantified by flow cytometric analyses in the bone marrow of this PCa mice models at day 25. [file 12916_2023_3094_MOESM2_ESM.zip › additional file 2/Fig.S3R4.pdf]
